# Sepsis-induced lung fibrosis in baboons is reduced by the treatment with a complement inhibitor

**DOI:** 10.1186/cc10403

**Published:** 2011-10-27

**Authors:** F Lupu, H Zhu, R Silasi-Mansat, C Georgescu, N Popescu, G Peer, C Lupu, F Taylor, G Kinasewitz, J Lambris

**Affiliations:** 1 Cardiovascular Biology Research Program, Oklahoma Medical Research Foundation, Oklahoma City, OK, USA; 2 Department of Pathology, Oklahoma University Health Sciences Center, Oklahoma City, OK, USA; 3 Neuroscience Research Department, Mayo Clinic Jacksonville, FL, USA; 4 Department of Medicine, Pulmonary and Critical Care Division, Oklahoma University Health Sciences Center, Oklahoma City, OK, USA; 5 Department of Pathology and Laboratory Medicine, School of Medicine, University of Pennsylvania, Philadelphia, PA, USA

## Introduction

Pulmonary fibrosis is a major and common medical condition, characterized by progressive scaring and decline in lung function. Persistent inflammation and acute lung injury in response to sepsis are potential triggers of the fibrotic response. Recently, we have reported that *Escherichia coli *sepsis in baboons strongly induces procoagulant responses and affects the integrity of the lung. These effects are diminished by the treatment with compstatin, a C3 convertase complement inhibitor [1].

## Methods

Here we used the baboon model described [1] in conjunction with detailed gene expression analysis, as well as biochemical and histological assays to determine if *E. coli *sepsis triggered metabolic and signaling pathways related to lung remodeling and fibrosis, and whether complement inhibition could attenuate these pathways.

## Results

Microarray gene expression analysis shows that sepsis augments several fibrotic gene clusters in the lung as early as 24 hours post *E. coli *challenge. Immunochemical and biochemical analysis reveals enhanced collagen synthesis, induction of profibrotic factors and increased cell recruitment and proliferation. Compstatin treatment decreases sepsis-induced expression of extracellular matrix genes, including eight collagen genes. Sirius Red and immunofluorescence staining for procollagens 1 and 3 confirms the collagen deposition in the lung. Ingenuity^® ^pathway analysis of transcriptomics data shows that compstatin treatment reduces sepsis-induced expression of genes involved in fibroblast transformation and connective tissue production, cell chemotaxis, migration and proliferation (see Table 1). Immunocytochemistry and pathway-oriented transcriptomics and phospho-proteomics analysis reveal changes of multiple processes mediated by transforming growth factor beta (TGF-β), connective tissue growth factor and other TGF-β controlled proteins. Immunostaining for cell proliferation markers demonstrates that compstatin treatment strongly reduces cell proliferation in fibroblastic foci. Moreover, biochemical analysis shows decreased production in the compstatin-treated group of two chemokines responsible for fibrocyte recruitment (CCL2 and CXCL12) and of the type 1 tissue inhibitor of metalloproteases that controls extracellular matrix remodeling.

**Table 1:** 

	*E. coli*				*E. coli *+ CS T+5			
		
	Modified genes				Modified genes			
				
	Up	Down	Total	*P *value	Up	Down	Total	*P *value
Fibroblast transformation	32	10	42	1.41 × 10^-6^	12	69	81	1.09 × 10^-7^
Connective tissue disorder	133	59	192	2.62 × 10^-5^	92	341	434	3.1 × 10^-7^
Chemotaxis	39	13	52	5.0 × 10^-3^	31	92	123	1.98 × 10^-5^
Cell migration	95	40	135	2.11 × 10^-5^	80	250	330	9.36 × 10^-8^
Cell proliferation	187	70	257	2.67 × 10^-8^	118	466	584	5.83 × 10^-13^
Fibroblast proliferation	23	7	30	4.80 × 10^-3^	10	62	72	5.56 × 10^-6^

CS T+5, compstatin-treated animals at T + 5 hours.

## Conclusion

Our data demonstrate that bacterial sepsis initiates pulmonary collagen deposition, and complement inhibition effectively attenuates the fibrotic response. This suggests that complement inhibitors could be used for prevention of sepsis-induced pulmonary fibrosis.
